# Effects of Valence and Origin of Emotions in Word Processing Evidenced by Event Related Potential Correlates in a Lexical Decision Task

**DOI:** 10.3389/fpsyg.2016.00271

**Published:** 2016-03-02

**Authors:** Kamil K. Imbir, Tomasz Spustek, Jarosław Żygierewicz

**Affiliations:** ^1^Faculty of Psychology, University of WarsawWarsaw, Poland; ^2^Faculty of Physics, University of WarsawWarsaw, Poland

**Keywords:** valence, origin of emotion, duality of mind, word processing, lexical decision task

## Abstract

This paper presents behavioral and event-related potential (ERP) correlates of emotional word processing during a lexical decision task (LDT). We showed that valence and origin (two distinct affective properties of stimuli) help to account for the ERP correlates of LDT. The origin of emotion is a factor derived from the emotion duality model. This model distinguishes between the automatic and controlled elicitation of emotional states. The subjects’ task was to discriminate words from pseudo-words. The stimulus words were carefully selected to differ with respect to valence and origin whilst being matched with respect to arousal, concreteness, length and frequency in natural language. Pseudo-words were matched to words with respect to length. The subjects were 32 individuals aged from 19 to 26 years who were invited to participate in an EEG study of lexical decision making. They evaluated a list of words and pseudo-words. We found that valence modulated the amplitude of the FN400 component (290–375 ms) at centro-frontal (Fz, Cz) region, whereas origin modulated the amplitude of the component in the LPC latency range (375–670 ms). The results indicate that the origin of stimuli should be taken into consideration while deliberating on the processing of emotional words.

## Introduction

This paper contributes to research on event-related potential (ERP) correlates of emotional word processing. There are several dimensions of emotional quality of stimuli charged with affect (Osgood et al., 1957). The valence (Kissler et al., 2007, 2009; Herbert et al., 2008; Schacht and Sommer, 2009a,b), arousal (e.g., Cuthbert et al., 2000; Schupp et al., 2000, 2004; Dillon et al., 2006; Fischler and Bradley, 2006) and concreteness (e.g., Kanske and Kotz, 2007; Barber et al., 2013; Palazova et al., 2013) of words modulate ERP correlates of word processing. Recently, we showed that the origin of an affective state also influences emotional word processing (Imbir et al., 2015a). In this study we used a standard lexical decision task (LDT) to compare the processing of lexically meaningful words and formally similar (readable, multi-syllabic) pseudo-word stimuli (Imbir et al., 2015b). We were interested in two aspects of processing. Firstly, we decided to compare word and pseudo-word reading conditions to find differences attributable to lexical processing (e.g., Münte et al., 1997; Bentin et al., 1999). Secondly, we investigated differences in the brain correlates of processing of emotional words caused by involuntary - implicit - semantic processing. Based on the emotion duality model (c.f. Jarymowicz and Imbir, 2015), we hypothesized that emotional word processing would be influenced by the origin of an emotional state included in word meaning.

### The Emotion Duality Model

The diversity of human emotions is as great as the number of people in the world (Kagan, 2007). Science searches for ways to organize this diversity and tries to explain what constitutes an emotion, rather than simply describing their diversity. For example the constructionist point of view (e.g., Russell, 2003) claims that the diversity of emotions derives from the human mind’s creation of subjective emotional states based on *core affect* – a description of the state of an organism in terms of pleasantness (valence) and activation (arousal) – with engagement of specific mechanisms (e.g., attributions, constructions of objects and situations evoking emotions and so on). Each state contributes to subjectively perceived experience.

*Duality of mind* theories (e.g., Gawronski and Creighton, 2013) offer a different perspective on emotional diversity, contrasting automated and controlled processes. The recently proposed emotion duality model (Jarymowicz and Imbir, 2015) is based on the concept of duality of mind. It postulates the existence of two separate evaluative mechanisms, namely the automatic evaluating system (AES), based on direct evaluations of external stimulation, and the reflective evaluating system (RES), based on cognitive appraisals (c.f. Imbir et al., 2015a).

The concept of AES processing is based on the biological value criterion proposed by Damasio (2010), which stipulates that all organisms are driven to preserve their lives. Biological value criterion does not require language to appear; evaluations just happen, and they immediately influence the organism’s state of mind, motivation and behavior. For example, sweetened water has a universal biological appeal because sugar is a source of energy, which is required to maintain life. Some stimuli have positive or negative biological significance (e.g., tasty food, appealing sexual partner, warm and sunny weather, snakes, nasty smells or decomposing corpses and so on). Our reaction to such stimuli may be immediate, because they have occurred repeatedly in human evolutionary past. Furthermore, these situations have shaped reproduction probability (making it less or more likely), thus organisms have evolved to react to biologically significant stimuli by approaching or avoiding them (Damasio, 2010).

Reflective evaluating system processing is based on verbalization (Jarymowicz and Imbir, 2015). Language is a crucial tool that allows our minds to organize stimuli effectively and gives us a temporal perspective (Rolls, 2000; Damasio, 2010) which includes both an imagined past and future. Language also increases our ability to distinguish emotional states, and to some extent allows us to modify our default biological responses. For example, fatty food may be evaluated as tasty and pleasant but, an individual who is trying to lose weight may adjust his or her evaluation to reflect this goal. It is possible that if one’s motivation to lose weight is strong enough fatty food would be judged repulsive, rather than attractive. Use of linguistic evaluation criteria (Reykowski, 1989) relies on reflective processing, which is further based on propositional thinking (Strack and Deutsch, 2004, 2014). This type of thinking requires effortful processing, but gives us an opportunity to modify automatic behavior in order to achieve a goal. Verbalization and use of language is such a frequent human activity that we can easily forget about or neglect its importance (c.f. Rolls, 2000). The constructionist theories of emotion (Russell, 2003), the appraisal theories (e.g., Scherer, 2005) and the emotion duality approach (e.g., Jarymowicz and Imbir, 2015) argue that language and controlled processes are crucial for understanding the diversity of emotions.

**FIGURE 1:**
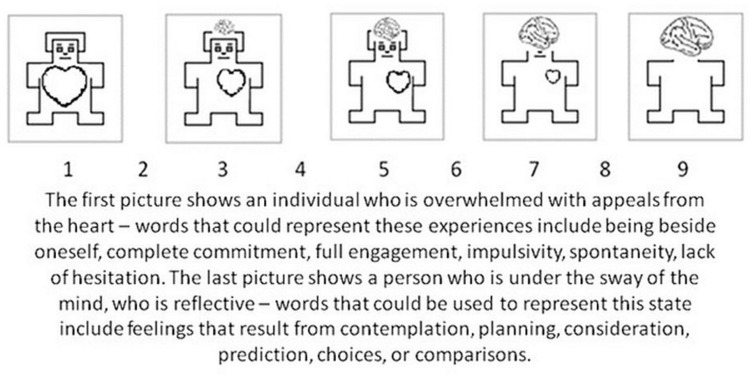
**Self-Assessment Manikin (SAM) scale and the description developed for use in measuring the origin dimension in the Affective Norms for Polish Words study (Imbir, 2015)**.

The origin of an emotional state (AES or RES) may modulate its influence on behavior. The basic assumption underlying this study is that origin, as a property of systems for processing emotional experience, is represented in language. This is supported by claims of the lexical hypothesis formulated in a field of personality psychology (e.g., Crowne, 2007). The lexical hypothesis states that characteristics important for people’s lives will eventually become a part of their language, and should be represented in words and lexical structures describing them or associated with them. We think that the crucial distinction between automatic and controlled processing is likely to be represented in language (Imbir, 2015) and that even states which can arise without an associated linguistic representation will have a verbal label (e.g., pain). This was the rationale for our studies concerning affective norms for Polish words (Imbir, 2015, submitted). To capture AES and RES processing we used a Self Assessment Manikin (SAM) scale with a heart and mind metaphor (Imbir, 2015). This metaphor compares and contrasts (1) purely emotional processing “from the heart,” which is immediate and automatic, with (2) careful consideration and reflection “from the mind.” In order to clarify the SAM scale the additional descriptions of its meaning were presented at the top of the scale. This gave us more confidence that the participants would use the scale as intended and it resulted in very good reliability of estimations (c.f. Imbir, 2015). **Figure 1** presents the SAM scale and the descriptions provided to participants to clarify its meaning.

It is worth comparing the proposed origin dimension to the established dimension of concreteness since both, to some extent, describe the complexity of stimuli. Concrete stimuli are stimuli in the physical world that we can touch or see, especially stimuli that can be easily represented by a picture or a word. Abstract stimuli are states that do not exist in the physical world (e.g., ideas or processes). These cannot be achieved without language or some sort of symbolic representation. It is especially hard to show them in one unambiguous picture. Automatic responses are immediate responses to stimulation. As in the case of a concrete stimuli, our mind labels AES emotional states, but they are able to appear even without language. Reflective responses are the product of RES-based appraisal and are based on language; they cannot occur without language. This makes concreteness and origin similar, but to verify this we analyzed affective ratings of 4900 Polish words of every grammatical class (Imbir, under review). In that study, participants assessed, among other variables, the origin of the state evoked by reading a given word, and the concreteness of the words. Origin and concreteness were only weakly correlated across the entire sample of words (*r* = -0.299, *p* < 0.001); they had only 10% common variance, which is low given the similarities mentioned above. On this basis we claim that origin and concreteness are distinct constructs, and that it is worth investigating effects of origin, controlled for variability in concreteness.

We think also that the origin of an emotional state is an important modulator of cognitive processes. This expectation is based on duality of mind theories (e.g., Gawronski and Creighton, 2013) which assert that many processes including decision making (e.g., Epstein, 2003; Kahneman, 2003; Darlow and Sloman, 2010), attitude formation (Gawronski and Creighton, 2013) and choices (Kahneman, 2011) are influenced by two types of ‘mind.’ The emotion duality model is an attempt to describe the outcomes of emotional processes for cognitive processes. The origin of stimuli was found to be related to ability to maintain cognitive control (Imbir and Jarymowicz, 2013), to modulate attention (Imbir, 2013) and to influence ERP correlates of word processing (Imbir et al., 2015a). Subjective significance, supposed to be the type of activation characteristic for reflective processes, was shown to eliminate the increase in response latencies caused by arousal in a modified Stroop task (Imbir, 2016). Taken together these results prompted us to investigate whether words with different origins would be processed differently in way that would be reflected in electroencephalography (EEG) correlates.

### Stages of Visual Processing of Emotional Words

The processing of visual words comprises several different stages (Bentin et al., 1999): the visual encoding of letters, translation of the letter shapes into a sequence of graphemes and orthographic patterns and, finally, activation of lexical and phonological structures and their meanings. Existing models of word processing (e.g., Rastle, 2007) do not include an affect as a salient factor in word recognition. Recently some evidence for an interaction between emotional valence and concreteness of a stimulus in word processing has emerged (e.g., Palazova et al., 2013). Effects of visual word processing on the N200, N400, FN400, and P600 (or LPC) ERP components have been found.

The N200 component evident in posterior locations is thought to be a manifestation of orthographic processing (e.g., Nobre et al., 1994) which distinguishes meaningful words, pronounceable pseudo-words and unpronounceable non-words from other complex, non-orthographic stimuli.

In anterior locations the N400 component is considered to be a manifestation of an unexpected event in speech perception and reading, such as when the last word in a sentence is not consistent with the sentence structure or meaning (e.g., Kutas et al., 1984). This N400 component is elicited only by words or pseudo-words that follow phonological rules (Bentin et al., 1999) and has been found even in paradigms which use isolated printed or spoken words rather than sentences as stimuli (e.g., Bentin et al., 1993). In frontal and central locations the N400 component represents higher order processing of semantic meaning and the word recognition stage of processing (c.f. Kutas and Federmeier, 2011) and it has also been shown to be sensitive to multiple lexical variables (Barber and Kutas, 2007) such as orthographic, phonological and word class information.

The FN400 component is similar to N400, but located in frontal regions (Kutas and Federmeier, 2011). Curran (2000) investigated the effects of familiarity and recollection of words. He showed that familiarity influenced the ERP waveform in the 300-500 ms interval in frontal location (FN400) whilst recollection was related to LPC (400-800 ms) in posterior locations. Further studies have demonstrated that FN400 is not specific to word processing (Voss and Paller, 2007); it also occurs in response to abstract geometric shapes which are being rated for perceived meaningfulness. In Voss and Paller (2007) study stimulus familiarity was manipulated as number of repetitions. Only meaningful shapes elicited an FN400 component, suggesting that this component is connected with conceptual priming rather than familiarity (Voss and Paller, 2007; Kutas and Federmeier, 2011).

The P600 component found in posterior locations is sometimes termed (e.g., Palazova et al., 2013) a late positive complex (LPC), and is considered to be a manifestation of deeper processing of word meaning (for a review see: Citron, 2012). The P600 component was found to be sensitive to processing difficulty and stimulus valence. Citron et al. (2011) reported that neutral words that were less salient than comparison valenced words elicited a larger LPC response during a LDT. Other higher order processes, such as perception of stimulus relevance (Fischler and Bradley, 2006), behavioral performance in the task (Polich, 2007), processing of self-referential (e.g., “my happiness”) versus other-referential (e.g., “his/her success”) words (Herbert et al., 2010, 2011) and processing of words differing in origin of emotional state (Imbir et al., 2015a) also modulated LPC amplitude.

It is worth emphasizing that emotional words are processed differently from other, more salient emotional stimuli. For example, although we might expect that attending to emotional stimuli would influence early ERP components - as processing faces or emotional scenes does – whereas in fact emotional word processing appears to modulate later ERP components associated with semantic analysis (Palazova et al., 2013). To conclude, existing evidence suggests that the emotional content of a word modulates the N200 amplitude (in studies of emotional word processing the N200 component is often referred to as the early posterior negativity, EPN), as well as in the N400 and P600 (or LPC) components (for a review see Citron, 2012).

### The Origin of the Emotional State as a Factor Modulating the Processing of Words

Our revious ERP study of word processing (Imbir et al., 2015a) have shown that the amplitude of early (EPN) and late (LPC) ERP components is modulated by both the valence and origin of words. In that study participants performed an odd-ball task. They were instructed to decide whether a word presented to them had negative or positive connotations, and to avoid responding to a predefined standard word. Independent component analysis (ICA) revealed a specific independent component with dipolar fronto-occipital topography that corresponded to the modulation of EPN. It had a higher absolute amplitude for positive words than for negative words. LPC amplitude was more positive for stimuli of reflective rather than automatic origin. ICA also revealed a dipolar independent component located in the left parietal region (direction left-parietal – right-frontal) which had a higher absolute amplitude (in the 437-570 ms window) in the case of emotional stimuli which engaged the automatic system rather than the reflective system. This difference was not observed in processing of neutral words. The emotion-related difference in the left parietal independent component described above (Imbir et al., 2015a) was not specific to concreteness differences.

### Aims and Hypotheses

The aim of this study was to investigate how the valence and origin of stimuli influenced implicit, involuntary lexical processing of verbal stimuli in a LDT. Previous studies of word processing in a task which explicitly demanded lexical processing (Imbir et al., 2015a) convinced us that this would extend understanding of emotional word processing mechanisms. Our argument is that origin is an important property of words and that it is processed independently of valence.

We expected words to elicit stronger ERP responses than pseudo-words in time ranges considered sensitive to word processing (N200, FN400 and LPC components). Considering words conditions only we did not expect effects of valence and origin on N200 component as this component is thought to reflect early processing, and hence orthographic rather than semantic processing (e.g., Nobre et al., 1994). This is in contrast to tasks involving explicit processing of words, where EPN effects related to allocation of visual attention have been reported (c.f. Citron, 2012). We expected to find differences in involuntary semantic processing (not related to instructions or to the task) with respect to the FN400 and LPC components. We expected that valence-related differences would occur sooner (in the FN400 time range) whilst origin-related differences would occur later (in the LPC time range).We also expected that origin effects would be asymmetrical (c.f. Imbir et al., 2015a) and evident mainly in the posterior regions of the left hemisphere.

## Materials and Methods

### Participants

Thirty-two individuals (women = 15; men = 17) aged from 19 to 26 years (*M* = 21.5, *SD* = 1.63) participated in the study. They were students at various Warsaw colleges and universities and participated voluntarily for a small reward. All of the participants were right-handed, native Polish speakers with normal or corrected-to-normal vision. Participants provided verbal, informed consent to participation; we did not collect written consent as we had assured the participants of anonymity. Oral consent was provided in the presence of at least two members of the laboratory and documented by them in a research diary. This procedure was suggested by the bioethical committee which approved the research. We did not collect any personal data from our participants. The design, experimental conditions and consent procedure for this study were approved by the bioethical committee of the Maria Grzegorzewska University.

### Design

The study consisted of two stages: first we searched for differences between the processing of words and pseudo-words. At this stage, we applied two-factor repeated measures analysis of variance (stimulus type × location) to successive time intervals. We assumed that activity in intervals where such differences are detectable is relevant to specific aspects of word processing. During the second stage responses to words were analyzed further by means of a three-factorial design: origin (automatic, A; no particular origin or mixed origin, 0; reflective, R) × valence (negative, Neg; neutral, Neu; positive, Pos) × scalp location. Combinations of conditions are referred to using the concatenation of their abbreviations, e.g., Neg_0 represents a negatively valenced word of no particular origin. We controlled for variability in arousal, concreteness, length and frequency of words and for participants’ handedness, gender and use of medication.

### Linguistic Material and its Properties

#### Emotionally Charged Words

The linguistic material consisted of a set of nouns divided into nine groups of 15 words; groups were matched for arousal, concreteness, length, and frequency, but differed with respect to valence and origin, yielding a 3 (valence) × 3 (origin) factorial manipulation. The selection of words was based on a previous study (Imbir, under review) in which the valence, origin, arousal and concreteness of 4905 Polish words were assessed by at least 50 participants (25 women), studying at various Warsaw universities. The database study used the same methodology as a previous study to define the properties of 1586 Polish words (Imbir, 2015). For the different levels of valence and origin we selected words as follows, level 1: score at least 1 *SD* below mean (Neg or A); level 2: score within 0.5 *SD* of the mean (Neu or 0); level 3: score at least 1 *SD* above the mean (Pos or R). All selected words had scores within 0.5 *SD* of the mean for arousal and concreteness. The selection procedure also ensured that the groups were matched for frequency and word length (NoL). Word frequency estimates were based on occurrence in a database of online Polish texts (Kazojć, 2011) and represent the number of times each word appeared in the database. The distribution of frequencies in this database was positively skewed so word frequency data were natural logarithm (LN) transformed to permit use of parametric statistics.

To check that our manipulations of word properties operated as intended we conducted 3 (valence) × 3 (origin) ANOVAs for each measured dimension. We found the predicted group differences in valence and origin ratings and an absence of group differences in arousal, concreteness and frequency. Neu and 0 words appeared to be about one letter shorter than the words in other groups. The full results of these analyses can be found in Appendix 1 (Word properties). **Table 1** presents means and standard deviations for word properties for all groups. See Appendix 2 for a complete list of selected words and their properties.

**Table 1:** Descriptive statistics (*M*, *SD*) for groups of words used in factorial manipulation.

	*M*	*(SD)*	*M*	*(SD)*	*M*	*(SD)*	Origin category	*M*	*(SD)*
Valence	3.50	(0.36)	5.02	(0.56)	6.71	(0.35)	Automatic	5.07	(1.39)


Origin	4.45	(0.53)	4.58	(0.37)	4.33	(0.70)		4.45	(0.55)


arousal	4.37	(0.49)	4.15	(0.55)	4.28	(0.80)		4.27	(0.62)


concreteness	4.31	(1.15)	3.95	(0.74)	4.48	(1.20)		4.24	(1.05)


NoL	7.20	(2.65)	7.47	(1.96)	7.40	(2.41)		7.36	(2.31)


Ln_freq	5.21	(1.91)	5.65	(2.03)	5.73	(2.28)		5.53	(2.04)


Valence	3.37	(0.36)	5.19	(0.54)	6.38	(0.32)	Control (0)	4.98	(1.32)


Origin	5.41	(0.31)	5.49	(0.30)	5.36	(0.35)		5.42	(0.32)


arousal	4.15	(0.23)	4.12	(0.67)	4.04	(0.51)		4.11	(0.49)


concreteness	4.05	(1.12)	3.96	(1.32)	4.17	(0.74)		4.06	(1.06)


NoL	6.47	(2.03)	5.27	(1.33)	6.93	(2.02)		6.22	(1.92)


Ln_freq	5.48	(2.28)	5.97	(1.27)	6.61	(2.02)		6.02	(1.92)


Valence	3.66	(0.35)	5.30	(0.39)	6.49	(0.40)	Reflective	5.15	(1.23)


Origin	6.46	(0.30)	6.63	(0.41)	6.63	(0.56)		6.57	(0.43)


arousal	4.32	(0.49)	3.93	(0.47)	4.03	(0.36)		4.10	(0.46)


concreteness	4.17	(1.13)	4.09	(1.17)	4.41	(1.07)		4.22	(1.11)


NoL	7.07	(1.75)	6.27	(1.62)	7.20	(2.27)		6.84	(1.91)


Ln_freq	5.42	(1.37)	6.53	(1.79)	6.01	(1.22)		5.99	(1.52)


	
**Valence category**	**Negative**		**Neutral**		**Positive**			**Total**	
					


Valence	3.51	(0.37)	5.17	(0.50)	6.53	(0.38)	Total	5.07	(1.31)


Origin	5.44	(0.92)	5.57	(0.92)	5.44	(1.09)		5.48	(0.97)


arousal	4.28	(0.42)	4.07	(0.56)	4.12	(0.58)		4.16	(0.53)


concreteness	4.18	(1.11)	4.00	(1.08)	4.35	(1.01)		4.18	(1.07)


NoL	6.91	(2.15)	6.33	(1.86)	7.18	(2.20)		6.81	(2.09)


Ln_freq	5.37	(1.85)	6.05	(1.72)	6.12	(1.89)		5.85	(1.84)




### Pseudo-Words

Pseudo-words were taken from the Polish Pseudo-words List (Imbir et al., 2015b), a dataset consisting of a large number of randomly generated pseudo-words stimuli assessed by competent judges as fulfilling the criteria for pseudo-word stimuli. The 135 pseudo-words used in this study were selected from a list of 864 pseudo-word stimuli that were positively verified by all of five judges and matched the 135 word stimuli as closely as possible with respect to length (number of letters). Appendix 1 (Table A1) presents the list of pseudo-words used in the experiment.

### Procedure

Participants were informed about the aim of the experiment and nature of the EEG measurement. We encouraged them to maintain a comfortable posture and control their eye blinks. The protocol provided 3-s breaks for normal blinking every 10 trials, as well as two longer breaks, whose duration controlled by the participant, for rest and adjustment of posture. The long breaks occurred every 270 trials.

The task was to read stimuli as they appeared in the middle of the screen and to classify them as words or pseudo-words by pressing tagged keys on the keyboard. The content and latency of responses were recorded. A single experimental block comprised 135 words and 135 pseudo-words; this block was repeated three times. Words and pseudo-words were displayed in random order in all blocks. Trials proceeded as follows: (1) fixation point displayed for 500 ms; (2) stimulus displayed until participant responds; (3) blank screen displayed for randomly varied interval between 1000 and 1100 ms.

### EEG Materials

#### Apparatus

Stimuli were displayed on a standard PC monitor (LCD display; 15-inch diagonal). A second PC was used for monitoring and recording EEG data. Stimuli and EEG data were synchronized using a custom-made hardware trigger. EEG activity was recorded from 19 electrode sites, Fz, Cz, Pz, Fp1/2, F7/8, F3/4, T3/4, C3/4, T5/6, P3/4, O1/2, referenced to linked earlobes, grounded on the clavicle and with impedances of 5 kΩ or less. The signal was acquired using a Porti7 (TMSI) amplifier with a sampling frequency of 256 Hz.

#### Offline EEG Signal Processing

Offline analysis was performed in Matlab^®^ with the EEGLAB toolbox (Delorme and Makeig, 2004). The statistical tests were implemented using the appropriate R procedures (R Development Core Team, 2008, available from: http://www.R-project.org). The signal was zero-phase filtered with Butterworth low- and high-pass filters (second order: corresponding to 12 dB/octave roll-off, with half amplitude cut-off frequencies of 30 and 0.1 Hz respectively), and with an IIR notch filter to remove line noise at 50 Hz. To suppress activity common to most of the data channels, the data were re-referenced to common-average montage. Epochs from 300 ms pre-stimulus to 1000 ms post-stimulus were extracted and baseline-corrected (baseline data taken from -200 to 0 ms).

The data were visually inspected to exclude error and artifact trials (e.g., eye blinks or muscle activity) the mean number of artifact-free trials eliciting a correct classification response was as follows: word trials *M* = 338 (*SEM* = 7); pseudo-word trials *M* = 348 (*SEM* = 7). Paired sample *t*-tests revealed a difference between trial types, *t*(31) = 4.17, *p* < 0.0003. For further analysis of ERP data in the words × pseudo-words design, randomly selected trials of the more numerous stimulus type were removed on a per subject basis to achieve a sample in which there were equal numbers of error-free trials of each type (word; pseudo-word) for each subject, a procedure suggested by Thomas et al. (2004). After this equalization procedure the mean number of trials of each type was 335 (*SEM* = 7).

The mean number of trials in per word condition was 37.5 (*SEM* = 0.3). The Friedman test for replicated block design did not indicate differences in the average number of trials per condition for the valence groups with origin as a blocking variable [χ*^2^*(2) = 3.9, *p* = 0.1], or for the origin groups with valence as a blocking variable [χ*^2^*(2) = 4.7, *p* = 0.1].

## Results

### Behavioral Measures

The distributions of behavioral measures, i.e., response time and response accuracy were non- normal according to a Shapiro–Wilk test for all word and pseudo-word categories. Differences between levels of experimental factors were therefore assessed with non-parametric tests.

#### Response Times

Response times were longer for pseudo-words (*M* = 934 ms, *SEM* = 44 ms) than for words (*M* = 776 ms, *SEM* = 28 ms). The Wilcoxon signed-rank test yielded *V* = 523, *p* < 5e-09. There were no effects of experimental factors on response latency for word trials. The Friedman test for replicated block design did not indicate differences in response times for valence groups with origin as a blocking variable [χ*^2^*(2) = 2.7, *p* = 0.2], or for origin groups with valence as a blocking variable [χ*^2^*(2) = 0.4, *p* = 0.8]. The mean response time was 777 ms (*SEM* = 10 ms).

#### Response Accuracy

There were more errors on pseudo-word trials [*M* = 4.1% (*SEM* = 0.3%)] than word trials [*M* = 3.1% (*SEM* = 0.4%); the Wilcoxon signed-rank test yielded *V* = 390, *p* < 0.02]. **Table 2** shows mean response accuracy and standard error for word trials in the valence × origin design. The Friedman test for replicated block design revealed an effect of valence group with origin as a blocking variable [χ*^2^*(2) = 38.8, *p* < 1e-08], and an effect of origin group with valence as a blocking variable [χ*^2^*(2) = 14.8, *p* < 0.001].

**Table 2:** Percentage correct responses in (*M* and *SEM)* for each stimulus category.

	Neg	Neu	Pos	Total
A	96.8 (0.6)	96.0 (0.7)	98.4 (0.4)	97.1 (0.4)
0	91.0 (1.3)	96.0 (0.7)	99.1 (0.3)	95.4 (0.6)
R	98.0 (0.4)	97.8 (0.4)	99.0 (0.3)	98.3 (0.2)
Total	95.3 (0.6)	96.6 (0.4)	98.8 (0.2)	96.9 (0.3)


Using the Wilcoxon pairwise test with the Holm correction for multiple comparisons we demonstrated that Pos words were more likely to be classified correctly than Neg (*p* < 2e-8) and Neu words (*p* < 2e-6). Zero words (control condition) were less likely to be classified correctly than A words (*p* = 0.05) and R words (*p* < 0.003). A *post hoc* test using the Wilcoxon rank sum test with the Holm correction showed that the highest number of errors was associated with Neg_0 words, more errors were with Neg_0 words than in all other conditions except Neu_A words.

### Electrophysiological Data

#### Time Windows and ROIs Selection

**FIGURE 2:**
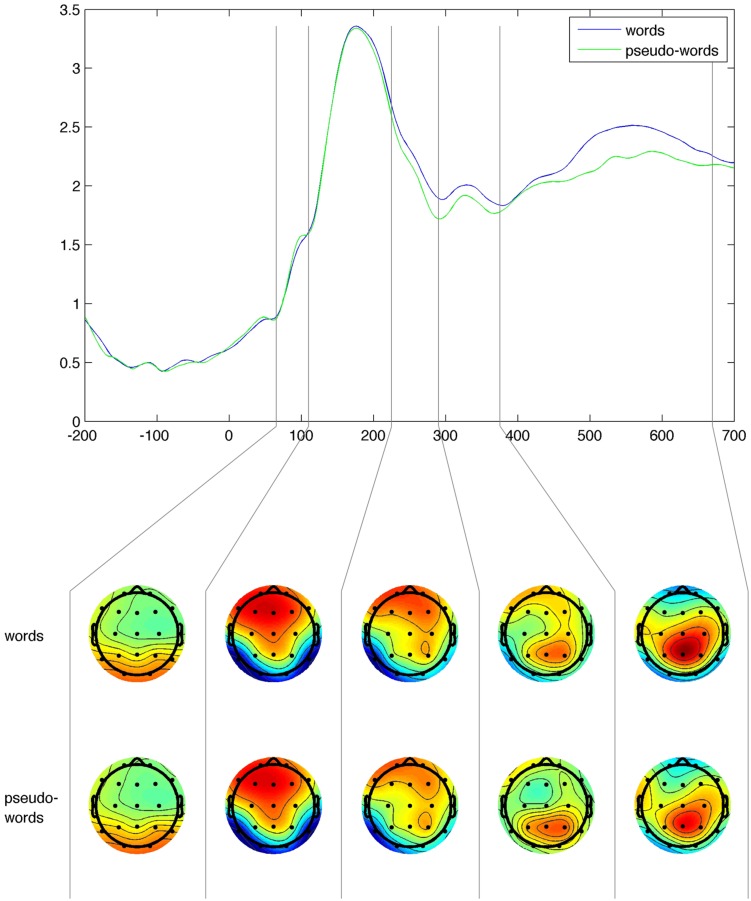
**Global field power **(upper plot)** and mean amplitude topographies (two **bottom rows**) for word and pseudo-word stimuli.** The vertical lines in the upper plot mark the boundaries of time windows.

Event-related potential data were analyzed for the following time windows: 65–110, 110–225, 225–290, 290–375, and 375–670 ms, based on the global field power (GFP) curve (**Figure 2**). GFP is computed as spatial standard deviation, and quantifies the sum of electrical activity over all electrodes at a given time point. The latencies of GFP maxima indicate the latencies of evoked potential components (Lehmann and Skrandies, 1980; Skrandies, 1990). **Figure 2** shows that the amplitude topographies for the first two time-windows are very similar for words and pseudo-words. In the remaining three time windows there are differences between words and pseudo-words with respect to amplitude and distribution. In the fourth time window word stimuli produced larger amplitude responses in the frontal regions than pseudo-words. The time windows used in the analysis correspond also to these that were assigned a potential role in word processing (Bentin et al., 1999; Kutas and Federmeier, 2011).

We selected five regions of interest (ROIs): left frontal (Fp1 and F7), centro-frontal (Fz and Cz), right frontal (Fp2 and F8), left parietal (C3 and P3) and right parietal (C4 and P4). Signal amplitudes in these regions were averaged across the corresponding electrode sites. Those regions are specific to components of interest (FN400, LPC: c.f. Voss and Paller, 2007). Based on previous findings (Imbir et al., 2015a) we expected that origin effects would be lateralized and therefore investigated left, right and central ROIs. We used an ROI approach rather than analyzing individual components at specific sites and time-windows suggested by the literature in order to not to bias the data analysis by subjective choices. The precise location of certain components varies between studies (e.g., Kutas and Federmeier, 2011) for methodological reasons. Our approach was based on the assumption that averaging activity from different sites in one ROI would allow us to identify the maximal for a given area response without subjectively choosing for analysis a single electrode. Also use of this approach was motivated by an incorporation of origin dimension, not examined earlier, thus potentially resulting in amplitude changes in different sites. On the other hand consideration of all individual electrodes would augment the problem of multiple comparison.

#### Analysis of Differences between Words and Pseudo-Words

We carried out separate repeated measures ANOVAs (stimulus type × ROI) on mean amplitude for each time window. Amplitude was measured as the mean amplitude (averaged over the duration of the time window) as this is more robust against electrical noise and latency jitter than maximum amplitude in a given time window (Luck, 2005). There was a main effect of ROI in all time windows, but since only interactions with the location factor are meaningful in the case of average referenced data this finding is not discussed further. For the time windows where there was an effect of stimulus type *post hoc* analysis using Holm-corrected paired sample *t*-tests was used to identify the regions in which the effect was significant. Interactions between ROI and stimulus type are detailed below for each time window. No stimulus type effects were observed for time windows 65-110 ms and 110-225 ms.

##### 225-290 ms time window

There was a simple effect of stimulus type [*F*(1,31) = 17.84, *p* < 0.0002] and an interaction between stimulus type and ROI [*F*(4,124) = 2.89, *p* < 0.03]. *Post hoc* analysis revealed that response amplitude was more positive for words (*M* = 0.84 μV (*SEM* = 0.21 μV) than for pseudo-words *M* = 0.53 μV (*SEM* = 0.21 μV) at the centro-frontal ROI [*t*(31) = 4.59, *p* < 0.0004].

##### 290-375 ms time window (corresponding with N400 or FN400)

There was a simple effect of stimulus type [*F*(1,31) = 64.09, *p* < 5e-9] and an interaction between stimulus type and ROI [*F*(4,124) = 14.82, *p* < 7e-10]. *Post hoc* analysis showed that all frontal ROIs words elicited more positive amplitudes than pseudo-words, but at the left-parietal ROI this pattern was reversed. The details are presented in Appendix 1 (Table A2).

##### 375-670 ms time window (corresponding with LPC)

There was a simple effect of stimulus type [*F*(1,31) = 8.17, *p* < 0.008] and an interaction between stimulus type and ROI [*F*(4,124) = 9.89, *p* < 6e-7]. *Post hoc* analysis showed that at the centro-frontal, left parietal and right parietal ROIs words elicited more positive amplitudes than pseudo-words, but at left frontal and right frontal ROIs the opposite pattern was observed. The details are presented in Appendix 1 (Table A3).

#### Word Properties

**FIGURE 3:**
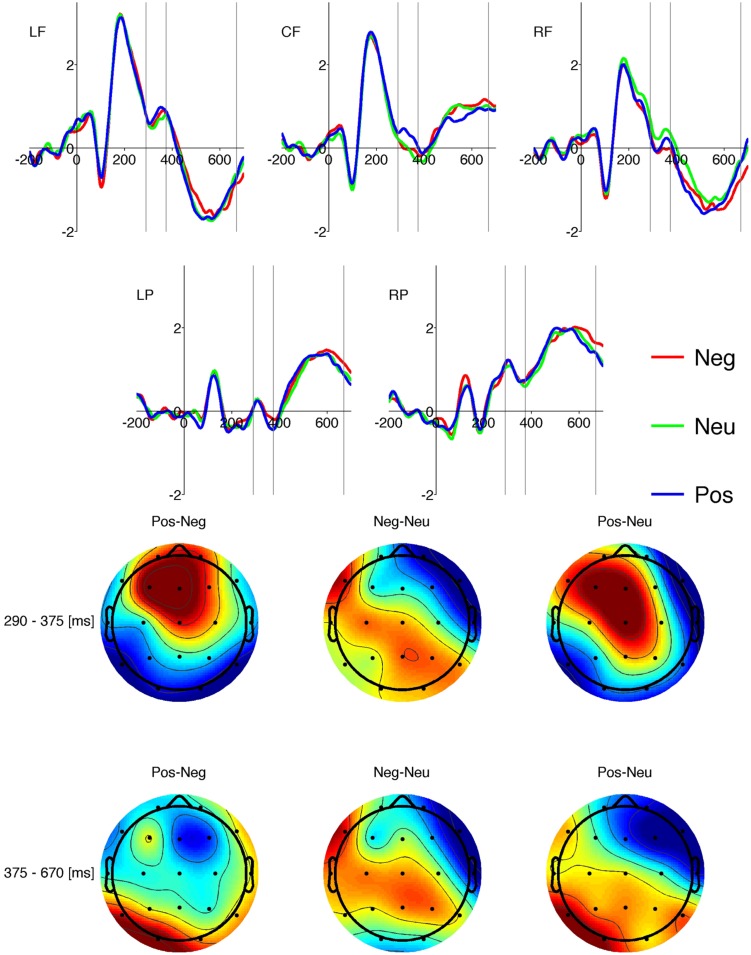
**Upper plots show grand average amplitudes of ERPs for different levels of valence (red: negative; green: neutral; blue: positive) at the five ROIs (LF: left frontal; CF: centro-frontal; RF: right frontal; LP: left posterior; RP: right posterior).** Vertical lines indicate the two time windows of interest. Lower plots show the topographical distribution of differences in amplitudes between the valence levels displayed above each plot. The color scale is red for positive and blue for negative value of the difference. Upper row show data for the 290-375 ms time window, lower row shows data for the 375-670 ms time window.

**FIGURE 4:**
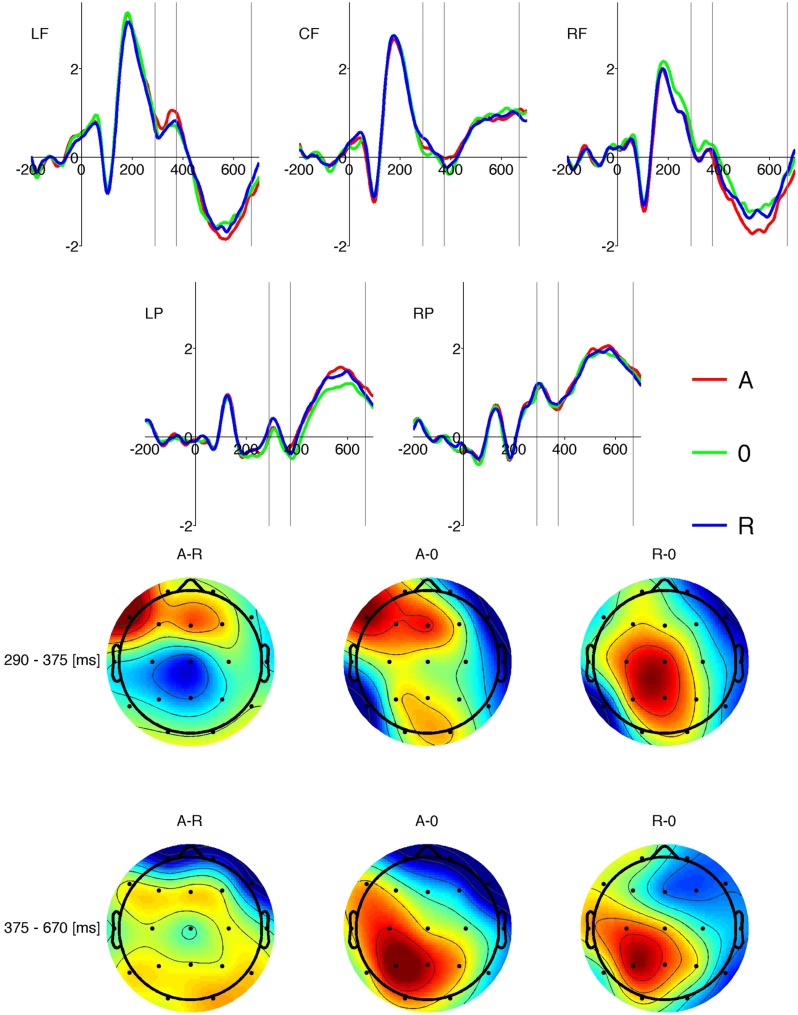
**Upper plots show grand average amplitudes of ERPs for different levels of origin (red: automatic; green: non-specific; blue: reflective) at the five ROIs (LF: left frontal; CF: centro-frontal; RF: right frontal; LP: left posterior; RP: right posterior).** Vertical lines indicate the two time windows of interest. Lower plots Show the topographical distribution of differences in amplitudes between the origin levels displayed above each plot. The color scale is red for positive and blue for negative value of the difference.Upper row show data for the 290-375 ms time window, lower row shows data for the 375-670 ms time window.

We carried out separate three-factor repeated measures ANOVAs (valence × origin × ROI) on mean amplitude data for each of the three time windows in which there were differences between responses on word and pseudo-word trials. For significant effects two-way analysis of variance was performed at each ROI followed by *post hoc* analysis using Holm-corrected paired samples *t*-tests. Only reliable effects in each time window and ROI are reported below. The results are illustrated as ERP time courses in **Figures 3** and **4.**

**Figure 3** shows than in the 290-375 ms time window there was a clear positive deflection in the centro-frontal ROI in response to stimuli with Pos valence but not Neu or Neg valence. The same patterns is observable in the topographical distribution of contrast potentials.

**Figure 4** illustrates an interesting origin-related difference in the time course of the response in right-frontal and left-parietal ROIs in the 375-670 ms time window. The topographical distribution of amplitude contrast reveals that the differences between A and R stimuli and 0 stimuli follow a dipolar pattern. These observations are corroborated by the statistical analysis reported below.

##### 225-290 time window

No reliable effects were observed.

##### 290-375 time window (corresponding with N400 or FN400)

There was a three-way interaction between valence, origin and ROI [*F*(16,496) = 2.77, *p* < 0.0003]. There was a main effect of valence at the centro-frontal ROI [*F*(2,62) = 6.44, *p* < 0.003]. *Post hoc* analysis revealed that this was due to higher amplitude responses to Pos stimuli [*M* = 0.31 μV, *SEM* = 0.14 μV] than to Neu stimuli [*M* = -0.05 μV, *SEM* = 0.15 μV; *t*(95) = 3.2, *p* < 0.004] and Neg stimuli [*M* = -0.02 μV, -*SEM* = 0.14 μV; *t*(95) = 3.44, *p* < 0.003].

There was an interaction between valence and origin for the right-frontal ROI [*F*(4,124) = 4.43, *p* < 0.003]. *Post hoc* analysis showed that Neu_0 stimuli elicited more positive amplitudes than Neg_A stimuli [*t*(31) = 5.17, *p* < 0.0005], Pos_0 stimuli [*t*(31) = 3.81, *p* < 0.03], Neg_R stimuli [*t*(31) = 3.64, *p* < 0.04] and Neu_R stimuli [*t*(31) = 3.53, *p* < 0.05]. The amplitudes for each condition are given in Appendix 1 (Table A4).

##### 375-670 time window (corresponding with LPC)

**FIGURE 5:**
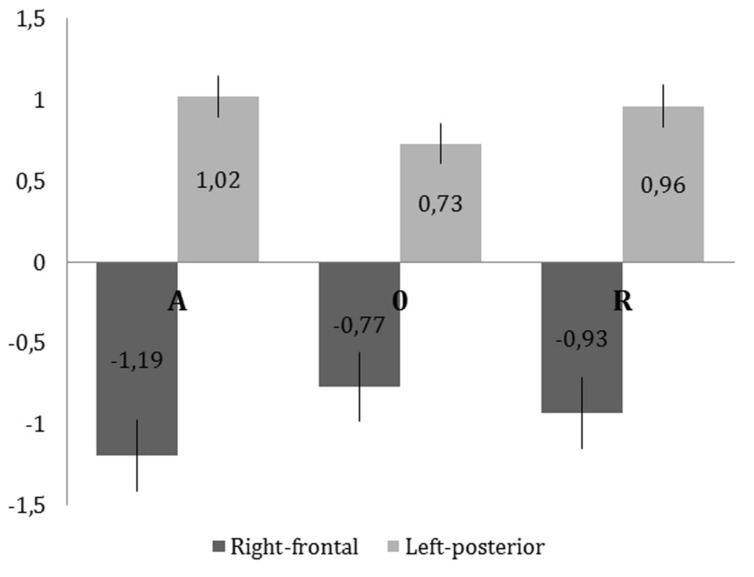
**Mean amplitudes for each level of origin in the right frontal (dark) and left posterior (light) ROIs in the 375-670ms time window.** The vertical lines represent *SEM*.

There was an interaction between origin and ROI [*F*(8,248) = 2.46, *p* < 0.014]. There was a main effect of origin at the right frontal [*F*(2,62) = 5.13, *p* < 0.01] and left parietal [*F*(2,62) = 6.39, *p* < 0.003] ROIs. **Figure 5** shows that responses in the left parietal and right frontal ROIs show similar origin-related changes although the polarity of the responses is reversed, which is the signature of a dipolar pattern. *Post hoc* paired sample *t*-tests showed that at the left posterior ROI responses to A and R stimuli were more positive than responses to 0 stimuli [*t*(95) = 3.27, *p* < 0.005 and *t*(95) = 2.80, *p* < 0.012 respectively]. At the right frontal ROI only the difference between A and 0 was significant [*t*(95) = 3.05, *p* < 0.01]. The higher error in amplitude data rendered the differences between 0 and R non-significant. This result is visualized in **Figure 5.**

## Discussion

Our study provides an orthogonal comparison of the impact on word processing of two properties of words, valence and origin. We controlled variability in factors such as arousal, concreteness, frequency and word length. Our pseudo-word stimuli were generated carefully and were matched in length with the word stimuli.

### Behavioral Results

Response times were longer for pseudo-words than for words, which suggests that decision making was more difficult in the case of pronounceable but meaningless stimuli. Contrary, there was no effect of valence or origin on response times in the LDT. This is consistent with a study by Barber et al. (2013) which found that concreteness but not valence affected response latencies, with abstract words eliciting faster responses than concrete words. Another study (Kanske and Kotz, 2007) also found that response latency was affected by concreteness, but not other word properties; however, in this study the opposite pattern was observed: concrete words elicited faster responses than abstract ones. From that reason, our results support the claim that origin and concreteness are distinct constructs (c.f. Introduction). Positive words were more likely to be classified correctly than negative and neutral words. Furthermore, words of no specific origin (control condition) were less likely to be classified correctly than automatic and reflective words. Further analysis revealed that this effect was due to poor classification of one stimulus category, namely negative words of no specific origin (c.f. **Table 2**). Barber et al. (2013) found a trend toward more accurate classification of concrete stimuli; concrete verbs were discriminated slightly more accurately than abstract words and pseudo-words. All electrophysiological data analyses were conducted using only data from accurate trials, thus we cannot attribute the obtained results strictly to the error rates of responses. Nevertheless those differences should be taken into account when interpreting the results.

### Differences between Words and Pseudo-Words

The amplitude of responses to words and pseudo-words differed in three time windows, namely the 225-290 ms, 290-375 ms, and 375-670 ms windows. This effect was modulated by electrode position. In the case of the 225-290 time window, differences were detectable in the centro-frontal ROI and words elicited larger - more positive – amplitude responses than pseudo-words. More research is needed to replicate this effect and evaluate conclusions that can be drawn from it. In the 290-375 ms time range identified as an N400 or FN400 component, the pseudo-words generated larger - more negative - responses than words in all frontal ROIs, whereas the opposite pattern of results (words elicit larger negative responses) was observed in the left parietal ROI.

In the 375-670 ms time range identified as a LPC component, words generated larger amplitude responses than pseudo-words at the centro-frontal, left parietal and right parietal ROIs; in the left and right frontal ROIs the reverse pattern was observed. The absence of differences between words and pseudo-words in the early ERP component (110-225 ms: N200) suggests that there were no orthographical or other formal differences between the word and pseudo-word stimuli used in our study (Nobre et al., 1994). The FN400 component responses suggest some kind of a surprise associated with the processing of pseudo-words of no semantic meaning in the frontal regions of the brain (Bentin et al., 1999), which may be related to the greater reading difficulty of pseudo-words; this suggestion is supported by the reaction time data. Finally, the LPC effect suggests that meaningful stimuli elicit deeper processing of word meaning (Citron, 2012). The results of comparisons between pseudo-words and words suggest the paradigm used was valid and confirms the stages of visual word processing identified in earlier studies (Bentin et al., 1999).

### Differences Related to Valence and Origin

#### The FN400 Component

There was a main effect of valence at the centro-frontal ROI for amplitudes in the 290-375 ms time range. Positive words elicited more positive amplitude than negative and neutral words. This effect was detectable at frontal ROIs, which is consistent with the word versus pseudo-word findings in this time range and suggests that activity in this time range should be interpreted as an FN400 component (Curran, 2000; Voss and Paller, 2007; Kutas and Federmeier, 2011) rather than an N400 component (Bentin et al., 1999; Kanske and Kotz, 2007; Kutas and Federmeier, 2011) or an EPN as in some LDT studies (Bayer et al., 2012). The FN400 component is thought to be related to semantic processing, especially the link between stimulus and meaning (Kutas and Federmeier, 2011). One would expect to detect valence effects on this component, because positive, negative and neutral valenced words are grouped semantically in the mind (Kanske and Kotz, 2007). Previous findings on concreteness are not consistent. Kanske and Kotz (2007) found that concrete nouns elicited a larger N400 response than abstract nouns but Palazova et al. (2013) reported no concreteness effect for verbs in the EPN time range. Given that valence is an intuitive dimension and should be processed sooner and more easily than other dimensions such as concreteness (Bayer et al., 2012; Palazova et al., 2013), we expected that FN400 amplitude would be more strongly influenced by valence than by other word properties. The data confirmed this prediction: we found that ERP responses varied according to valence.

In the right frontal ROI there was an interaction between valence and origin. Since Neu_0 words elicited larger amplitude responses than the four other valence-origin combinations it is worth inspecting word properties. All stimulus groups were matched for concreteness, arousing properties and frequency of appearance in language, but there were some group differences in word length. Words in the Neu_0 group were on average about one letter shorter those in other word groups (c.f. **Table 1**; Appendix 1). Shorter stimuli are expected to be processed in an easier way. In current study paradigm we may assume that in the right frontal ROI response amplitude is negatively associated with stimulus length (c.f. LPC discussion).

#### The LPC Component

The LPC component was found to be sensitive to concreteness in the case of both nouns (Kanske and Kotz, 2007) and verbs (Palazova et al., 2013). Abstract nouns and verbs elicited larger LPC amplitude responses than concrete nouns and verbs. In our study the amplitude in the LPC time range differed according to the origin of the word in two ROIs, namely the right frontal and left parietal regions. In the right frontal ROI automatic words elicited more negative amplitude responses than 0 words whereas in the left parietal ROI both automatic and reflective stimuli elicited more positive amplitude response than 0 words. The scalp location and pattern of differences in both ROIs suggest activation of a dipolar source; amplitudes at the same locations were affected by word origin in a task requiring explicit emotional processing (Imbir et al., 2015a). Alternatively these results could be interpreted as effects of different, temporally correlated processes; the right frontal ROI effect could be related to differences in stimulus length and the left parietal effect could be related mostly to differences in origin of words.

Comparing the ERP results and behavioral data shows that words of no specific origin (0 words) were classified less accurately than words with a specific origin (automatic and reflective words). This suggests that specified origin of an affective component of word make decisions easier (after controlling for potential effects of frequency of appearance, concreteness and arousing properties). Since opposite patterns were observed in the two described above ROIs it is hard to explain the results in behavioral terms. It is also important to consider the group difference in word length in our selection of stimuli. Automatic words were longer than 0 words (c.f. Linguistic materials properties and **Table 1**). In line with FN400 component findings for the right frontal ROI we predicted that shorter words would elicit larger amplitude responses than longer words. In fact this pattern is replicated in LPC time range in the same ROI. It should be noted that there was no difference in length between reflective and 0 words, just as there was no difference in the amplitude of responses to these stimulus types in the right frontal ROI. We can therefore conclude that responses in the right frontal ROI are sensitive to stimulus length.

In our previous studies using the odd-ball task to assess effects of the emotionality of words (emotional versus neutral valence character assessments; Imbir et al., 2015a), we found that the LPC response was influenced by the origin of emotion. Raw ERP amplitude was higher for words of reflective origin than words of automatic origin, but this effect could have been due to differences in the concreteness of the stimuli. ICA showed an independent component with a dipolar topography in left posterior locations for which amplitude was different only in the case of valenced (not differing from concreteness), but not neutral words (differing from concreteness). The amplitude of this component was larger for words of automatic origin than those of reflective origin.

Current study, which used a different task and different verbal stimuli, has confirmed our previous findings about location of region engaged in processing. It is worth highlighting that we found some differences in pattern of results. Words of automatic origin elicited more positive LPC responses than words of unspecified origin (this study) or words of reflective origin (previous study). This difference could be attributed to the judgment participants were required to make about stimuli in the two studies. In the earlier study (Imbir et al., 2015a) participants had to decide whether a word was emotional or neutral (explicit lexical processing of meaning), whereas in this study they had to classify stimuli as words or pseudo-words (implicit lexical processing). These tasks depend on underlying mental processes which differ with respect to depth and profile of analysis. Origin is an emotional property referring to whether processing engages the AES or RES. When we asked participants about the emotional quality of stimuli, the AES was associated with more crucial experiences (such as threats to life); these should be deeper and produce a higher amplitude than RES experiences. When we asked participants about lexical quality, the differences between systems could be less salient, but still the AES should attract more attention.

## Conclusion

We have presented data relevant to how lexical processing of words in a LDT is influenced by the valence and origin of stimuli. Valence influenced the amplitude of the FN400 component, whereas origin influenced the LPC. The study was designed carefully to avoid any potentially confounding factors biasing the results; we controlled for potential effects of concreteness, arousal, frequency of appearance and stimulus length in a factorial design. We found no reaction latencies differences across conditions, but we did find accuracy effects. Effects on the FN400 and LPC components in the right frontal region could be attributed to differences in task difficulty and stimulus length. Our findings on the effects of origin are consistent with an earlier study (Imbir et al., 2015a) indicating that left parietal regions are engaged in processing of stimulus origin. This suggests that the origin of emotional states is one of the factors that modulate late stages of word processing. The results of this study are important for understanding the role of complexity represented in stimuli evoking automatic or reflective originated emotional responses. We showed that this complexity is affective in character and not related to the concreteness of stimuli. What is more, this origin related complexity influences not only explicit, but also implicit processing of semantic stimuli.

## Author Contributions

All authors contributed to final version of the manuscript. Theoretical proposition: KI; Design: KI, JZ; Method (words): KI; Method (EEG measures) JZ, TS; Experimental procedure programming: TS, JZ; Experiment execution: TS, JZ; Statistical analyses: JZ, KI, TS; Results description: JZ; Results discussion: KI; Figures: JZ, TS, KI; Tables: JZ, KI.

## Conflict of Interest Statement

The authors declare that the research was conducted in the absence of any commercial or financial relationships that could be construed as a potential conflict of interest.

The reviewer DG and handling Editor declared their shared affiliation, and the handling Editor states that the process nevertheless met the standards of a fair and objective review
