# Supplementary material for: Effects of Valence and Origin of Emotions in Word Processing Evidenced by Event Related Potential Correlates in a Lexical Decision Task
Source: Front Psychol. 2016 Mar 2;7:271. doi: 10.3389/fpsyg.2016.00271 (PMC4773610; doi:10.3389/fpsyg.2016.00271)
Supplement: Supplementary file 2 [file Data_Sheet_2.DOCX]

**Appendix 1**

**Part 1: Words properties analyses results**

To ensure correct construction of the manipulation, we conducted 3 (valence levels) x 3 (origin levels) ANOVA analyses for each dimension measured. In the case of the valence ratings, they differed between valence levels: *F*(2,126) = 607.44, *p*< 0.001, *η^2^* = 0.91, but did not differ significantly between origin levels: *F*(2,126) = 1.88, *p* = 0.16, *η^2^* = 0.03. We did not find an interaction between valence and origin levels: *F*(4,126) = 2.09, *p* = 0.086, *η^2^* = 0.062. In the case of the origin ratings, they did not differ between valence levels: *F*(2,126) = 1.27, *p* = 0.28, *η^2^* = 0.02, but differed significantly between origin levels: *F*(2,126) = 254.55, *p*< 0.001, *η^2^* = 0.80. We did not find an interaction for origin assessments between valence and origin levels: *F*(4,126) = 0.5, *p* = 0.74, *η^2^* = 0.016. In the case of controlled variables, no statistically significant effects were found for arousal between valence levels: *F*(2,126) = 1.98, *p* = 0.14, *η^2^* = 0.02, or between valence and origin levels: *F*(2,126) = 1.44, *p* = 0.24, *η^2^* = 0.02. We did not find any significant interaction between valence and origin levels: *F*(4,126) = 0.5, *p* = 0.72, *η^2^* = 0.016. Regarding the concreteness ratings, we did not find statistically significant effects for valence levels: *F*(2,126) = 1.19, *p* = 0.31, *η^2^*= 0.02, or origin levels: F(2,126) = 0.4, *p* = 0.67, *η^2^* = 0.006. There was no significant interaction between valence and origin levels: *F*(4,126) = 0.12, *p* = 0.98, *η^2^*= 0.004.

Regarding the length of words used, we found no differences between valence levels: *F*(2,126) = 2.01, *p* = 0.14, *η^2^* = 0.03, but there was a difference between origin levels: *F*(2,126) = 3.48, *p* = 0.034, *η^2^*= 0.052. We did not find a significant interaction between valence and origin groups of levels: *F*(4,126) = 0.82, *p* = 0.52, *η^2^* = 0.025. Simple contrast analysis showed that the difference concerned words of an automatic origin versus words of no particular origin: *t*(132) = 2.62, *p* = 0.01, while remaining comparisons appeared insignificant. Automatically originated words were *M* = 7.3 (*SEM* = 0.3) letters long while words of no particular origin were *M* = 6.2 (*SEM* = 0.3) letters long. Finally, for the logarithm natural transformation of frequency of word appearance in the Polish language, no statistically significant effects were found for valence levels: *F*(2,126) = 2.3, *p* = 0.11, *η^2^* = 0.04, or for origin levels: *F*(2,126) = 1.0, *p* = 0.37, *η^2^* = 0.016. We found no significant interaction between valence and origin levels: *F*(4,126) = .44, *p* = 0.78, *η^2^*= 0.014.

**Part 2: Additional tables**

Table A1. Pseudo-words used in the current experiment.

| lum | czalka | gopliwyść | knem | minczanie | ośrotnaść | ratrokosanie | tarela |
| --- | --- | --- | --- | --- | --- | --- | --- |
| pek | dnań | greba | kodzykóbka | miniozer | pachurz | rondzialenie | trunkatka |
| adletaka | dojaszający | gwód | kojek | miuszydło | patercjał | rospikt | tubleca |
| atfas | dowan | halatka | komowinie | miwra | pedlona | rowdel | ubatowasie |
| azmata | dyltawor | hesztania | kopinan | moseta | pegła | rowpunta | ubok |
| bagół | ebir | hukory | kosfidant | msot | piebzy | ruczt | waczyn |
| blak | enerysura | hutor | ksab | mszowka | pilder | rulera | wajek |
| blirna | fadatazm | ikol | ksamra | nardekonie | pitenczał | rzesiodło | wcisąwanie |
| blodowka | flęta | izea | ksapa | obet | piwza | samat | weda |
| blutka | fluszracja | joskilia | ktew | odlarki | pleser | sewa | wezdnowienie |
| błyza | fukro | kabzeł | lęgiczbość | odpryw | płospek | silawka | wiekrz |
| botenka | gałsan | kakutka | łądka | odzardenie | pnug | skafka | zaupek |
| burdoter | gedzer | kasalach | maliołer | ołpa | podemlik | stodek | zmolsość |
| bzawo | gilfinta | kciny | mardzatek | onarzelie | posocyt | stowycja | zogałka |
| chorakłer | gingnena | kikucka | marpa | ontarz | powądasie | strzedło | zrenaksowinie |
| chowik | głanyca | kiszymówka | menodymka | oplanki | powojawka | szlejdor | żamoba |
| cywek | głyms | kladra | midion | ośręcz | pykon | szwodzik |  |

Table A2. The comparison of average amplitude related to words and pseudo-words for the time window 290-375 ms at different ROIs

| ROI | Amplitude [μV] | | *t*(31) | *p* |
| --- | --- | --- | --- | --- |
|  | Words  *M* (*SEM*) | Pseudo-words  *M* (*SEM*) |  |  |
| left-frontal | 0.71 (0.34) | 0.24 (0.3) | 3.96 | 0.001 |
| centro-frontal | 0.08 (0.22) | -0.61 (0.23) | 7.58 | 8e-8 |
| right-frontal | 0.14 (0.3) | -0.43 (0.3) | 4.56 | 0.0003 |
| left- parietal | -0.03 (0.16) | 0.2 (0.17) | -3.26 | 0.005 |
| right- parietal | 0.9 (0.25) | 0.79 (0.23) | 1.73 | 0.09 |

Table A3. The comparison of average amplitude related to words and pseudo-words for the time window 375-670 ms at different ROIs

| ROI | Amplitude [μV] | | *t*(31) | *p* |
| --- | --- | --- | --- | --- |
|  | Words  *M* (SEM) | Pseudo-words  *M* (*SEM*) |  |  |
| left-frontal | -0.94 (0.33) | -0.47 (0.31) | -3.0 | 0.01 |
| centro-frontal | 0.63 (0.25) | 0.15 (0.29) | 4.23 | 0.0007 |
| right-frontal | -0.97 (0.34) | -0.72 (0.35) | -1.46 | 0.15 |
| left-parietal | 0.91 (0.2) | 0.42 (0.2) | 4.36 | 0.0006 |
| right-parietal | 1.59 (0.22) | 1.28 (0.21) | 3.53 | 0.004 |

Table. A4. Amplitudes in μV for time window 290-375 ms at right frontal ROI*M* (*SEM*)

|  | Neg | Neu | Pos |
| --- | --- | --- | --- |
| A | -0.28 (0.37) | 0.18 (0.30) | 0.32 (0.35) |
| 0 | 0.27 (0.35) | 0.80 (0.33) | -0.18 (0.36) |
| R | 0.02 (0.36) | 0.08 (0.33) | 0.12 (0.34) |
